# Major tail proteins of bacteriophages of the order *Caudovirales*

**DOI:** 10.1016/j.jbc.2021.101472

**Published:** 2021-12-08

**Authors:** Maximilian Zinke, Gunnar F. Schröder, Adam Lange

**Affiliations:** 1 Structural Bioinformatics Unit, Department of Structural Biology and Chemistry, C3BI, Institut Pasteur, CNRS UMR3528, CNRS USR3756, Paris, France; 2 Institute of Biological Information Processing (IBI-7: Structural Biochemistry), Forschungszentrum Jülich, Jülich, Germany; 3 Physics Department, Heinrich Heine University Düsseldorf, Düsseldorf, Germany; 4 Department of Molecular Biophysics, Leibniz-Forschungsinstitut für Molekulare Pharmakologie (FMP), Berlin, Germany; 5 Institut für Biologie, Humboldt-Universität zu Berlin, Berlin, Germany

**Keywords:** phage, bacteriophage, phage tail, cryo-EM, NMR, solid-state NMR, crystallography, structural biology, protein dynamics, virus, AFP, antifeeding prophage, FN3, fibronectin type III, gp, gene product, GTA, gene transfer agent, Hcp, hemolysin coregulated protein, Ig, immunoglobulin, LPS, lipopolysaccharide, MTP, major tail protein, PVC, *Photorhabdus* virulence cassette, T6SS, type VI secretion system

## Abstract

Technological advances in cryo-EM in recent years have given rise to detailed atomic structures of bacteriophage tail tubes—a class of filamentous protein assemblies that could previously only be studied on the atomic scale in either their monomeric form or when packed within a crystal lattice. These hollow elongated protein structures, present in most bacteriophages of the order *Caudovirales*, connect the DNA-containing capsid with a receptor function at the distal end of the tail and consist of helical and polymerized major tail proteins. However, the resolution of cryo-EM data for these systems differs enormously between different tail tube types, partly inhibiting the building of high-fidelity models and barring a combination with further structural biology methods. Here, we review the structural biology efforts within this field and highlight the role of integrative structural biology approaches that have proved successful for some of these systems. Finally, we summarize the structural elements of major tail proteins and conceptualize how different amounts of tail tube flexibility confer heterogeneity within cryo-EM maps and, thus, limit high-resolution reconstructions.

Bacteriophages—or simply phages—comprise the group of viruses that infect prokaryotes, that is, eubacteria and archaea. These parasitic entities consist of a genome that is carried by a proteinaceous scaffold and, in some cases, lipids. For infection, their nucleic acids are shuttled into the cytoplasm of host bacteria for reproduction. Here, the bacterial protein biosynthesis machinery is seized to produce new phage particles, which are eventually released by host cell lysis to infect other bacterial cells. Because of this antibiotic mechanism, phages can be used to treat bacterial infection in a treatment called phage therapy. The current antibiotic crisis led to a rediscovery of phage therapy as demonstrated by recent compassionate uses or clinical studies (1, 2).

Tailed bacteriophages with a double-stranded DNA genome, which comprise the order of *Caudovirales*, account for 96% of all phages and form probably the predominant biological entity on earth—as suggested by metagenomics (3). This order is further subdivided into families by their tail morphology. *Podoviridae* and *Autographviridae* share a short knob-like appendage. *Siphoviridae*, *Demerecviridae*, and *Drexlerviridae* have a long, flexible, noncontractile tail, and *Myoviridae*, *Ackermannviridae*, *Chaseviridae*, and *Herelleviridae* have a long, rigid, and contractile tail with a sheath around a central tube. Before the International Committee on Taxonomy of Viruses 2019 release, *Caudovirales* were only subdivided into the families *Podoviridae*, *Siphoviridae*, and *Myoviridae* (4). Other tail-less morphotypes include polyhedral, filamentous, and pleomorphic—forming their own independent families (5).

## General architecture of tailed phages and their assembly and infection

**Figure 1:**
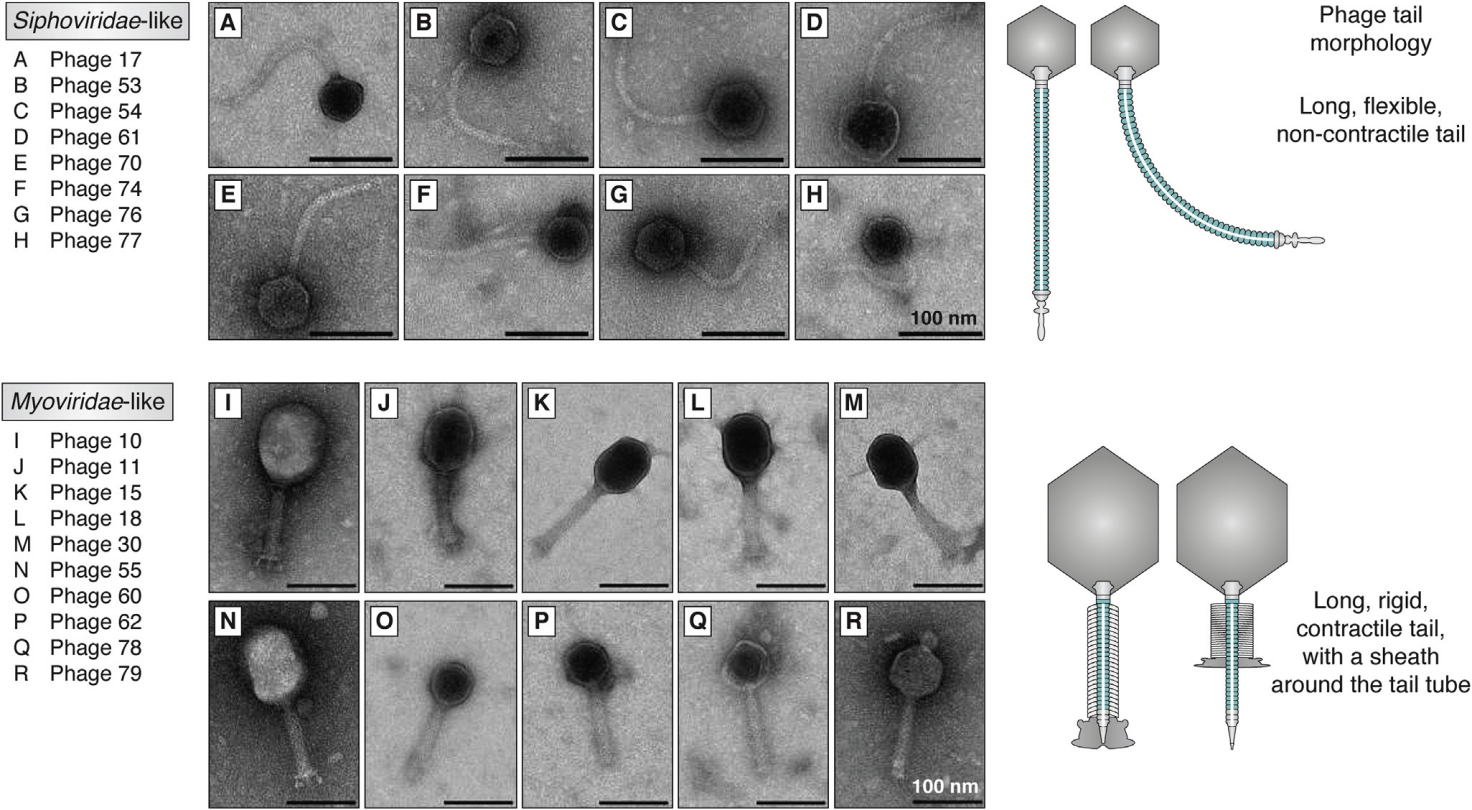
**Comparison of *Siphoviridae*-like and *Myoviridae*-like tail morphologies.***A*–*H*, *Siphoviridae*-like phages have a long, flexible, and noncontractile tail. *I*–*R*, *Myoviridae*-like phages have a long, rigid, and contractile tail with a sheath around the tail tube. The scale bar represents 100 nm. Reproduced with minor changes under a Creative Commons Attribution 4.0 International License (http://creativecommons.org/licenses/by/4.0/) from the study by Sørensen *et al.* (104).

**Figure 2:**
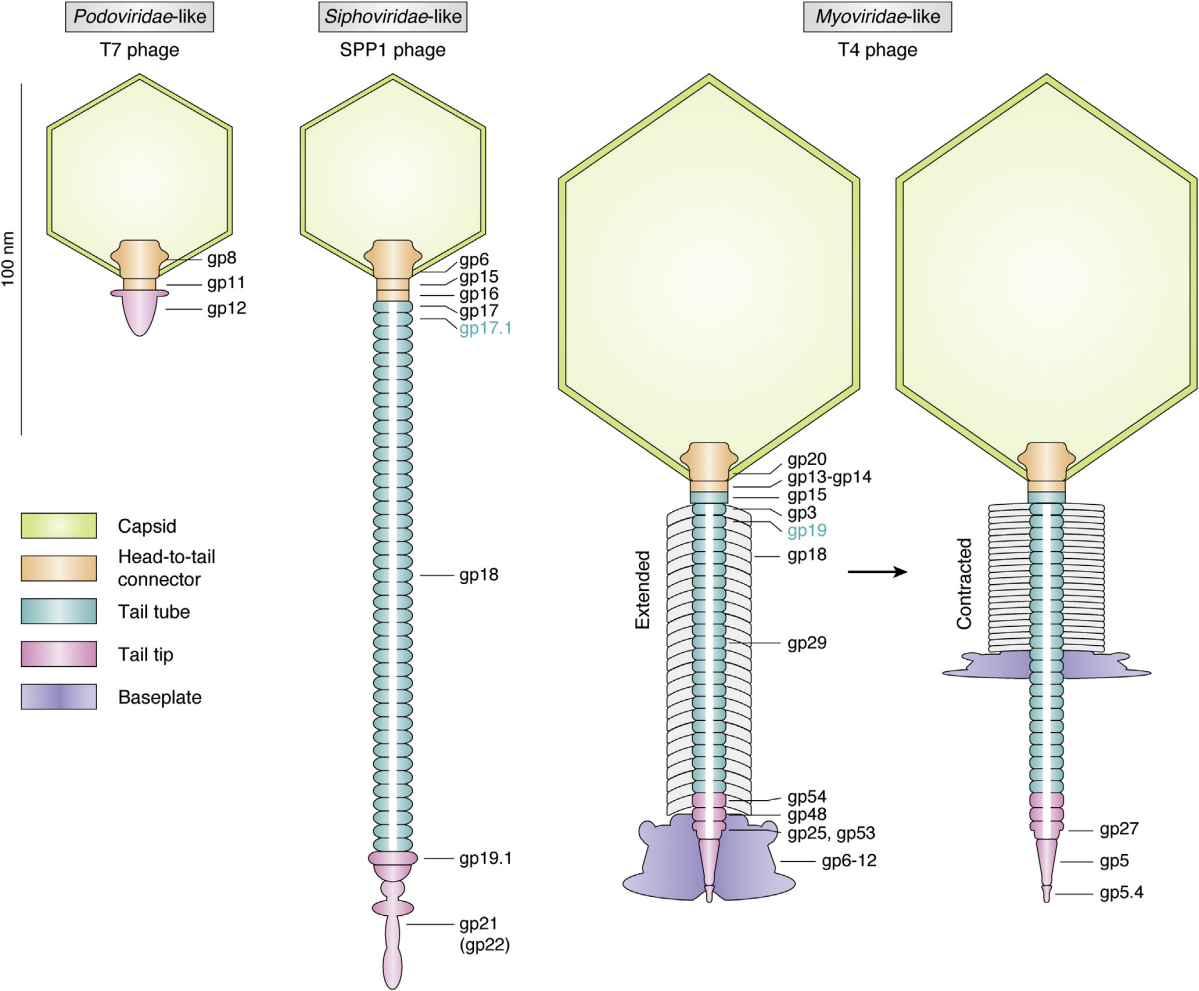
**Typical architectures of *Podoviridae*-like, *Siphoviridae*-like, and *Myoviridae*-like phage virions are visualized by cross sections through T7, SPP1, and T4 phages.***Myoviridae*-like virions consist of a capsid (*green*), a head-to-tail connector (*orange*), and a tail tip (*pink*) (6). *Siphoviridae*-like (15, 19, 24, 26, 27, 34, 105, 106) and *Myoviridae*-like (36, 37, 41, 44, 53) virions feature in addition a tail tube (*turquoise*) and in some cases—like T4 phage—a baseplate (*purple*) (45, 47). Furthermore, *Myoviridae*-like virions possess a sheath (gp18, *white*) assembled around the tail tube (*turquoise*), which contracts upon host cell recognition inducing a syringe-like piercing process (13, 59). The tail tubes are filled with tape measure proteins (gp18, gp29, *white*). Fiber proteins are not shown. Only noncapsid structural proteins are indicated.

To illustrate the general architecture of tailed bacteriophages, the structural proteins of one specific bacteriophage of each tail-morphotype group (*Podoviridae*-like, *Siphoviridae*-like, and *Myoviridae*-like) will be reviewed in detail—namely T7 phage (*Podoviridae*-like), SPP1 phage (*Siphoviridae*-like), and T4 phage (*Myoviridae*-like). It must be noted that within these groups, considerable structural differences—especially concerning the tail tips and capsid dimensions—exist (Fig. 1). However, this information is sufficient to put the major tail protein (MTP) into a structural context since the tail tube itself exists as a rather isolated entity. Also, capsid proteins as well as fiber and baseplate proteins will not be discussed in detail (Fig. 2).

T7 phage belongs to the *Autographviridae* family and is, thus, part of the *Podoviridae*-like tail-morphotype group. A central role is played by the portal protein gene product (gp) 8 that oligomerizes into a dodecameric complex forming a conical channel (6). This structure initiates procapsid assembly around itself—in a fashion that it winds up inserted in one vertex of the icosahedral unit (7, 8). The binding of the ATP-dependent packaging terminase to the portal complex initiates the active transport of DNA into the procapsid (9, 10). The release of the terminase and the completion of genome packaging reshape the prohead into the final icosahedral capsid. The formation of the tail is initiated by the assembly of the adaptor protein gp11 into a dodecameric ring, onto which the hexameric nozzle protein (gp12) assembles (11). The tail formation is completed by the binding of six gp17 fiber protein trimers, which are responsible for host cell recognition, onto the gp11–gp12 interface (12). This tail structure binds to the portal complex—closing off the capsid to prevent DNA leakage and completing the full virion (Fig. 2). The Myoviridae-like tail-morphotype phages do not possess an MTP. Host cell recognition commences by the binding of the distal half-fibers of gp17 to the core heptose region of *Escherichia coli* lipopolysaccharides (LPSs) (12, 13). Bacterial porins possibly act as primary receptors in this process (14). This binding is suggested to trigger a conformational change in the nozzle protein gp12 leading to its opening and the translocation of the viral DNA and core proteins (14). These core proteins are proposed to form a tunnel for DNA conduit across the bacterial membrane (12).

SPP1 phage belongs to the *Siphoviridae* family and is, thus, part of the *Siphoviridae*-like tail-morphotype group. The dodecameric portal protein gp6 sits on one vertex of the icosahedral procapsid of SPP1 phage. The maturation from procapsid to capsid occurs after the release of the scaffolding protein gp6 and the translocation of the viral DNA into the head (15, 16). Hereby, the DNA is ATP-dependently transported by the packaging terminase through the portal complex like a funnel (17). After capsid maturation, the portal complex is sealed to prevent DNA leakage by the binding of the hexameric ring–shaped adaptor protein complex gp15 and hexameric ring–shaped stopper protein complex gp16 onto the portal—forming the head-to-tail connector (18). A central role within the tail of SPP1 is played by the distal tail protein (Dit) gp19.1, which forms a hexameric ring. This structure acts as a hub for the distal tail adsorption function and the tail tube (19). The tail tube consists of 40 stacked hexameric rings of the MTP gp17.1 and its C-terminally extended variant gp17.1∗ assembled around the—possibly trimeric (20)—tape measure protein gp8, which was shown for TP901-1 phage to define tail tube length (21). gp17.1∗ is C-terminally extended by a ribosomal frameshift with a fibronectin type III (FN3) domain, an immunoglobulin (Ig) fold, and gp17.1 and gp17.1∗ occur in a ratio of 3:1 within the tail tube. However, it was shown that virions only containing gp17.1 are viable and infectious (22). The FN3 domain is proposed to transiently interact with carbohydrates of the cell wall of *Bacillus subtilis*, and, thus, allows for two-dimensional diffusion of the virions on the outer bacterial surface promoting the infection process (23). The top of the tail tube is tapered by a hexameric ring of the tail completion protein gp17 that enables the connection of the tail to the head-to-tail connector by binding to the stopper protein gp16 (24, 25). The distal tail adsorption function consists of the trimeric tail tip protein gp21, and possibly gp22, gp23, gp23.1, and gp24—however, their locations are unknown (26, 27). gp21 or a protein associated with it is responsible for the irreversible binding to the ectodomain of the YueB receptor of *Bacillus subtilis* inducing infection (28, 29). For λ phage, tail formation commences by the formation of an initiator complex, which includes the tape measure protein, assembly chaperones, the distal tail protein, baseplate proteins, and tail tip proteins. This complex acts as a platform for the MTP to polymerize onto the distal tail protein ring along the tape measure protein creating a helical tube replacing the assembly chaperones (30). After the tape measure protein is entirely engulfed by the tail tube, MTP oligomerization stops, and the tail is tapered by the tail completion protein and subsequently connects to the head-to-tail connector completing the full virion (31, 32). However, in contrast, the MTP of SPP1 phage (gp17.1) is able to self-polymerize in the absence of any additional protein (33). Hence, the tail formation process might be different. Binding to the YueB receptor leads to the dissociation of the tail tip, which subsequently primes the discharge of the tape measure protein out of the tail tube—where it is confined in a metastable state. Next, the diaphragm-like opening of the stopper protein gp16 culminates in the DNA ejection through the tail tube initiating infection (34). Tape measure proteins were proposed to play a role in pore formation in the host membrane for DNA translocation (35).

T4 phage belongs to the *Myoviridae* family and is, thus, part of the *Myoviridae*-like tail-morphotype group. The head assembly of this phage is more complex compared with the previous mentioned cases: The dodecameric portal protein gp20 forms a membrane-spanning initiation complex with gp40, which recruits 11 scaffolding proteins initiating procapsid assembly (36, 37, 38). The capsid shell consists of the major capsid protein gp23 and vertex protein gp24 that forms the fivefold vertices of the prohead. The scaffolding and capsid proteins are subsequently matured by proteolytic cleavage, which releases the procapsids from the membrane and provides space for the DNA (39). The DNA is translocated through the portal complex by the ATP-dependent terminase, which expands the procapsid into the final capsid (40). This expansion creates binding sites for the small outer capsid protein (gp soc) and the highly antigenic outer capsid protein (gp hoc) that decorate the surface of the capsid (41, 42). The architecture of the T4 phage capsid resembles a prolate icosahedron, that is, icosahedral ends and cylindrical equatorial middle sections (43). The portal complex is sealed off by the binding of the gp13–gp14 neck complex completing the head (44). Tail assembly commences by the association of the baseplate that consists of six wedges joined around a central tube (45, 46). The baseplate is appended at the proximal end by hexameric rings of gp48 and gp54, which act as hub for tail tube polymerization (47), and by gp27, gp5, and gp5.4 at the distal end forming the tail tip. The trimeric protein gp27 acts as a conduit for DNA passage, whereas the trimeric proteins gp5 and gp5.4 are involved in host membrane puncturing and host peptidoglycan hydrolysis (48, 49). The MTP gp19 polymerizes into 24 hexameric rings around the tape measure protein gp29 (50)—that is anchored within the baseplate—creating the tail tube, which is tapered at the proximal end by the hexameric tail tube terminator protein gp3 (51, 52). Subsequently, the sheath protein gp18 polymerizes helically around the tail tube (13). Binding of the hexameric tail completion protein gp15 onto gp3 and the last ring of the tail sheath completes the tail (53, 54). The full virion is completed by joining of the tail and the head-to-tail connector *via* gp15–(gp13–gp14) interaction. In addition, T4 phage possesses short and long tail fibers at the baseplate and a head whisker at the head-to-tail connector (55, 56). The binding of the long tail fibers to LPSs and OmpC of the outer membrane of *E. coli* triggers the short tail fibers to unwind from beneath the baseplate and irreversibly bind to LPS anchoring the baseplate onto the outer membrane (57). Rearrangements in the baseplate lead to the contraction of the tail sheath driving the tail tip through the outer membrane enabling digestion of the peptidoglycan layer and subsequent translocation of the tape measure protein gp29 and the viral DNA through the tail tube (47, 58). Possibly, the tape measure protein gp29 and/or gp27 form a pore in the inner membrane allowing for DNA conduit into the host cytoplasm (59).

## Bacteriophage tail–like systems

Gram-negative bacteria feature at least six known extracellular proteinaceous secretion systems, which are involved not only in the pathogenesis of higher organisms but also in the antagonistic defense between bacteria (60). The type VI secretion system (T6SS) resembles structurally a contractile *Myoviridae*-like phage tail—including a baseplate, a tail tube, and a sheath. However, its baseplate is not bound to the outer membrane to allow for its piercing from the outside. Instead, it is embedded in the inner membrane to allow for the piercing of the own outer membrane and the membrane of a target cell (61). In this fashion, it resembles a positional-reversed contractile phage tail (62). It is used to transport effector proteins, as opposed to nucleic acid in the case of phages (63). Within this secretion system, hemolysin coregulated protein (Hcp/tssD) resembles the MTP of bacteriophages forming hexameric rings that stack onto each other creating a hollow tube suitable for effector protein conduit into a host cell. Hcp polymerization requires the presence of the tail tip complex (62). This allows for the crystallization of Hcp hexamers and their structure determination by X-ray crystallography, as it will be discussed later.

Gene transfer agents (GTAs) are phage-like particles that are expressed by prokaryotes to shuttle random fragments of their genome to recipients giving rise to horizontal gene transfer. GTA genes are encoded within the genome of the producing cell. They are likely genetically related to phages and prophages but do never contain the full set of genes required to express themselves, that is, the ability to produce GTAs is never transmitted between bacteria in this process. Hence, they do not resemble an independent self-replicating unit unlike phages and prophages. All known GTAs have *Caudovirales*-like (tailed bacteriophages) architectures—possessing a tail, and, for the case of *Siphoviridae*-like and *Myoviridae*-like morphotypes, an MTP (64). Like the release of bacteriophages, GTA release requires the lysis of the host cell. This necessary sacrifice in addition to the lack of a preferential DNA replication before packaging puts the benefit of these systems for horizontal gene transfer in question, as they might just be defective prophages (65). However, it could be shown by differences in amino acid composition that likely more than half of all in bacteria-encoded prophages are instead GTAs (66).

Phage tail–like bacteriocins—widespread among bacteria—are bacterial defense machineries that kill competing bacteria by destroying their membrane potential. They are evolutionary related to bacteriophage tails and resemble capsid-less bacteriophages (67). Phage tail–like bacteriocins can be split into two groups based on their morphology: F-type tailocins consist of a flexuous and noncontractile tail—like *Siphoviridae*-like phages,whereas R-type tailocins consist of a rigid and contractile tail—like *Myoviridae*-like phages (68). Hence, both classes contain an MTP. Bacteriocins are assembled intracellularly upon SOS response. Like GTAs, bacteriocins are released by self-sacrificing producing cell lysis. Once released, they can bind to certain receptors on the surface of competing bacteria. Both type of tailocins form channel pores in the membrane of the target cell after receptor binding destroying the membrane potential and, thus, killing the cell. Like *Myoviridae*-like phages, R-type pyocins use a syringe-like mechanism involving the contraction of a sheath protein to pierce the cell membrane. As opposed to R-type pyocins (69, 70), there are no F-type tailocin tail tube structures published to date.

Phage-like protein-translocation structures are very similar to R-type pyocins—resembling capsid-less *Myoviridae*-type phages (71). However, they act by transporting and secreting toxins, as opposed to merely killing cells by membrane puncturing. They combine the morphology and need to be secreted of R-type pyocins with the effector protein transduction mechanism of T6SS (72). Hence, they contain an MTP that is environed by a sheath. Examples include the antifeeding prophage (AFP) from *Serratia entomophila* (73) and the *Photorhabdus* virulence cassette (PVC) (74). For AFP, it was shown that the toxin is transported in the lumen of the tail tube and, thus, replaces the tape measure protein in the mature AFP particle (75).

## MTP structure determination of phage tails and phage tail–like systems

In this chapter, the determined structures of the forementioned systems and their determination will be discussed in detail (Table 1). Herein, the focus will lie on those studies that describe MTP structures within the complex of the tail tube as opposed to monomeric structures, that is, monomers in the solvated state, or crystal structures. Historically, T6SS MTPs were first studied because of their ability to readily form crystals that feature hexameric unit cells—a subunit arrangement similar to the tail tube complex. First, *Siphoviridae*-like systems will be discussed starting with GTA as this system bears the simplest MTP structure. The underlying structural studies are summarized in Table 1. The intermolecular interfaces of the systems are detailed in Table 2.

**Table 1:** Determined structures of bacteriophage tail and bacteriophage tail–like MTPs

Tail system	MTP	Method	Protein Data Bank ID	Year	Resolution in Å
*Siphoviridae*-like					
GTA of *Rhodobacter capsulatus* (76)	Rcc01691	Cryo-EM	6TSV	2020	3.8
80α phage (20)	gp53	Cryo-EM	6V8I	2020	3.7
SPP1 phage (86)	gp17.1	NMR/cryo-EM	6YQ5	2020	1.8
λ-phage (81)	gpV	NMR/cryo-EM	6P3E	2019	5.4
YSD1 phage (85)	YSD1_22	Cryo-EM	6XGR	2020	3.5
T5 phage (80)	pb6	X-ray/cryo-EM	N/A	2017	6.0
*Myoviridae*-like					
T4 phage (47, 50)	gp19	Cryo-EM	5W5F	2017	3.4
AFP of *Serratia entomophila* (75)	Afp1	Cryo-EM	6RBN	2019	3.1
PVC (89)	Pvc1	Cryo-EM	6J0B	2019	2.9
Pyocin R2 (70)	Pa0623	Cryo-EM	6PYT	2020	2.9
T6SS from *Vibrio cholera* (88)	Hcp1	Cryo-EM	5OJQ	2017	3.7
T6SS from *Flavobacterium johnsoniae*	Hcp1	X-ray	6BDC	2017	2.5
T6SS from *Campylobacter jejuni* (97)	Hcp1	X-ray	6A2V	2018	2.6
T6SS from *Pseudomonas aeruginosa* (98)	Hcp3	X-ray	3HE1	2009	2.1
T6SS from *Salmonella typhimurium* (99)	Hcp2	X-ray	5XEU	2017	3.0
T6SS from *Burkholderia pseudomallei* (100)	Hcp1	X-ray	3WX6	2015	2.7
T6SS from *Acinetobacter baumanii* (101)	Hcp1	X-ray	4W64	2015	1.6
T6SS from *Edwardsiella tarda* (102)	EvpC	X-ray	3EAA	2009	2.8

Abbreviation: N/A, not available.

**Table 2:** Intermolecular interfaces in bacteriophage tail and bacteriophage tail–like systems between MTPs and, if present, the sheath

Tail system	Interaction surface in Å^2^ & ΔG in kcal/mol								
	Intraring		Interring					Sheath	Total
	i − (i ± 1)	i − (i ± 2)	i − j	i − (j + 1)	i − (j − 1)	I − (j + 2)	i − (j − 2)		
SPP1 phage	1372 & −6.5		856 & −12.8	600 & −2.2	34 & 1.2	99 & −1.7			5382 & −44.0
GTA	1535 & −18.4	60 & −1.3	137 & −2.2	464 & −3.5		77 & −1.3			4546 & −53.4
80α phage	1210 & −7.8		681 & −5.6	446 & −4.8		123 & −1.8			4920 & −40
λ phage	1587 & −17.5	5 & −0.1	68 & −0.3	340 & −3.4					4000 & −42.6
YSD1 phage	2519 & −18.7	125 & 0.4	165 & −2.3	566 & −4.2	62 & 0.4	75 & 1.5			7024 & −45.8
T5 phage	1340 & −12.1		763 & −4.0	210 & −3.2	46 & 0.4				4718 & −37.8
T4 phage	2149 & −16.4	120 & −0.6	389 & −4.9	392 & −5.0	296 & −2.2	140 & −1.5	169 & −2	N/A	7310 & −65.2
AFP	1547 & −15.8		243 & −5.7	514 & −4.6	484 & −3.6	99 & −0.6		782 & −4	5774 & −60.6
PVC	1577 & −24.4		243 & −4.8	532 & −5.7	507 & −3	85 & −1.4		186 & −0.5	5888 & −78.6
Pyocin R2	1965 & −22.3		573 & −6.6	380 & −0.9	234 & 0.3	71 & −0.4		711 & 3.8	6446 & −59.8
T6SS	1699 & −24.4		603 & −5.6	291 & −1.1	210 & −1.9	108 & 0.5		720 & 1.4	5822 & −65.0

**Figure 3:**
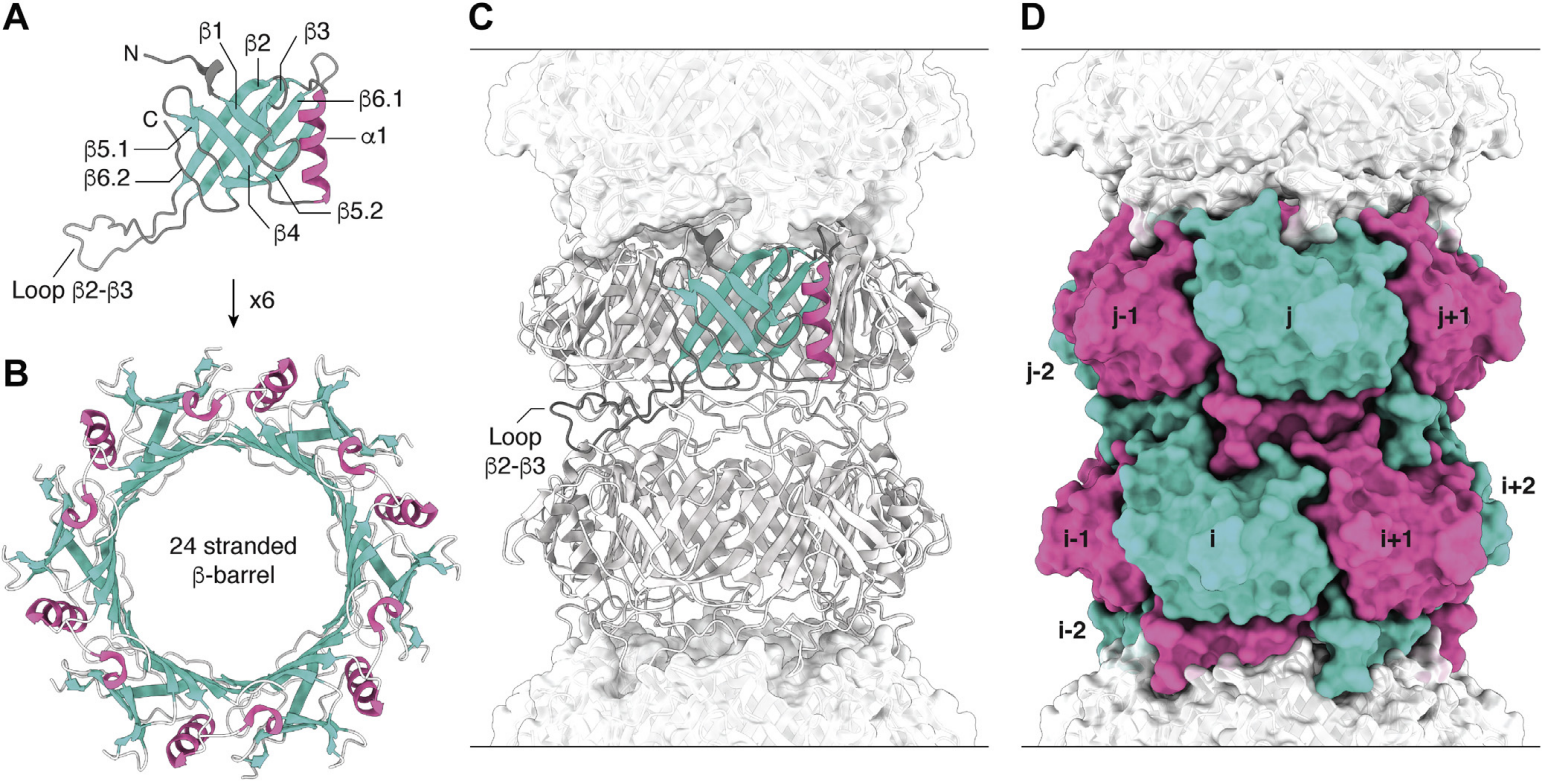
**Architecture of the tail tube of the gene transfer agent (GTA) of *Rhodobacter capsulatus*** (76) **as the most minimal MTP system.***A*, the MTP Rcc01691 consists of a β-sandwich–type fold (*turquoise*) flanked by an α-helix (*pink*) and the loop β2–β3. *B*, six MTPs form a hexameric ring by creating a 24-stranded β-barrel along β2–β3–β6.1–β5.2; herein the intermolecular interfaces are formed by β2–β5.2. *C*, these hexameric rings stack helically onto each other creating the hollow tail tube. Interring contacts are mostly mediated by the loop β2–β3 that folds onto the subjacent ring. *D*, nomenclature of subunits within two rings i and j. MTP, major tail protein.

Interface areas and solvation free energy gain were determined by the PISA Webserver (103). The nomenclature of intermolecular contacts follows the convention introduced in Figure 3. The total interface areas and solvation free energy gains are calculated by summing all contributions and multiplying them by two since all contacts occur twice per subunit in such homo-oligomeric complexes.

### GTA tail tube of *Rhodobacter capsulatus*—*Siphoviridae*-like

**Figure 4:**
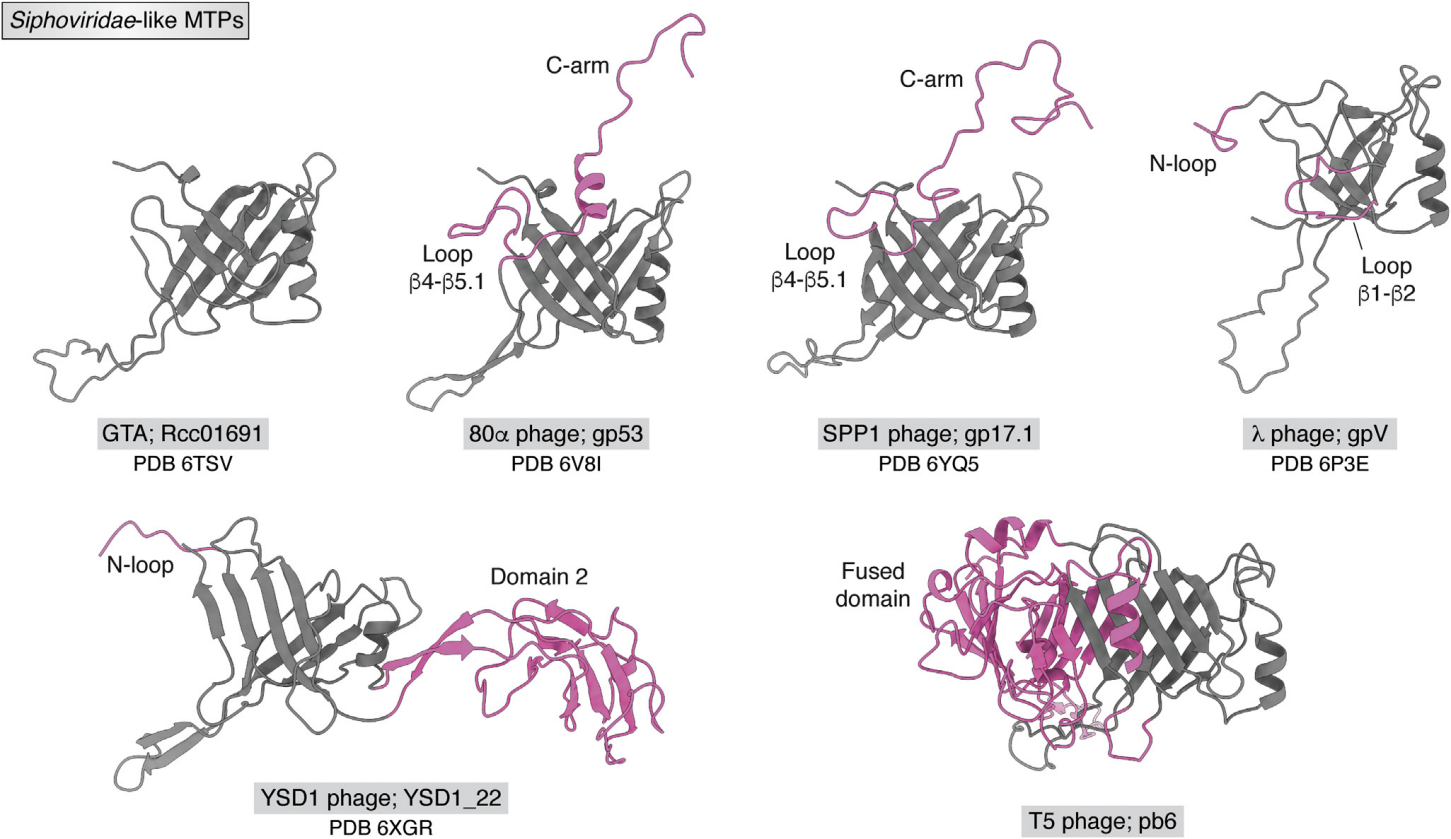
**Comparison of *Siphoviridae*-like MTPs.** GTA is used as the most basic MTP system. In comparison to it, additional domains or loops are colored in *pink*. MTPs are labeled with system name, protein name, and Protein Data Bank ID. Ig-fold–like domains of T5 phage and λ phage are not shown. The pb6 structure was available through personal communication with the authors. GTA, gene transfer agent; MTP, major tail protein.

**Figure 5:**
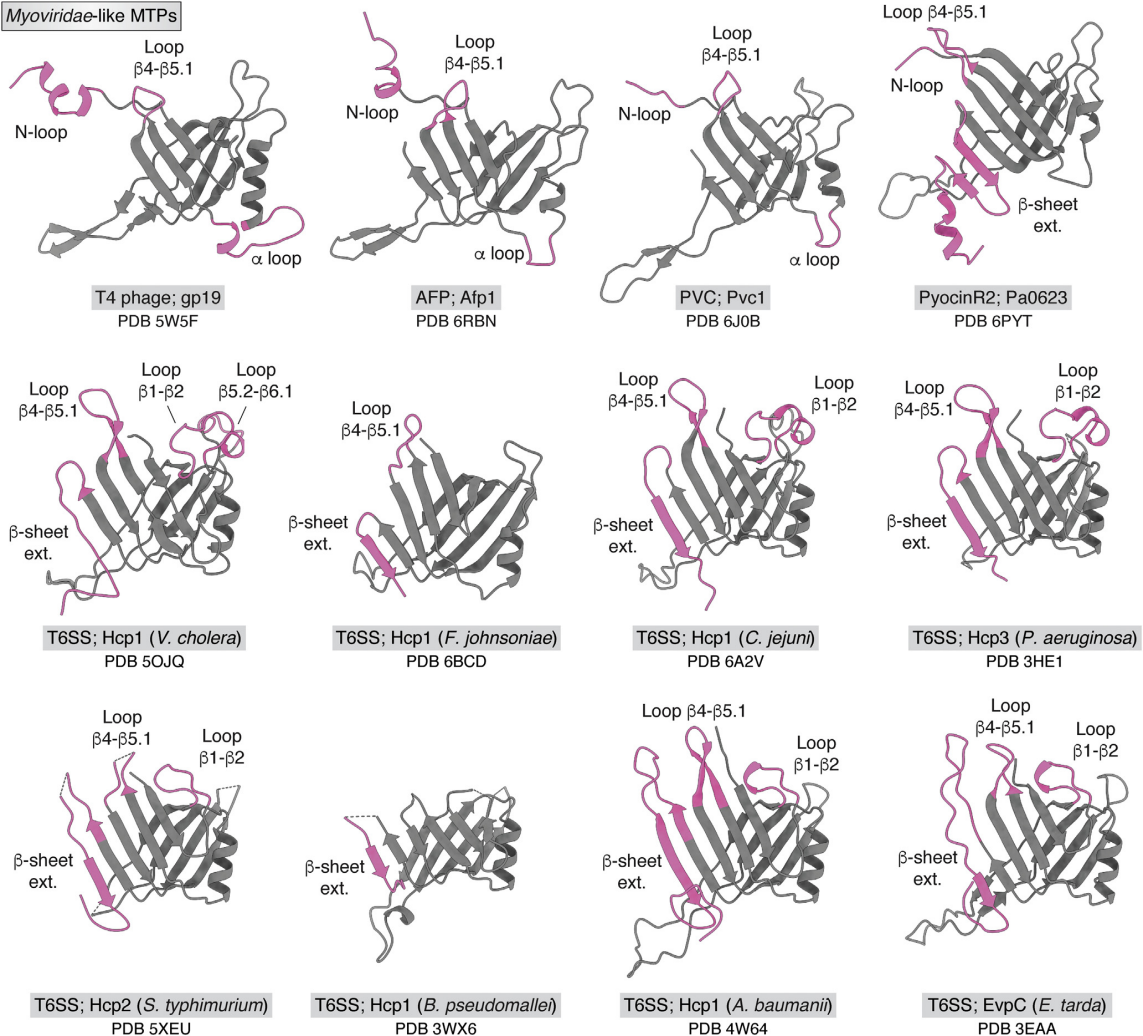
**Comparison of *Myoviridae*-like MTPs.** GTA is used as the most basic MTP system (Fig. 3). In comparison to it, additional domains or loops are colored in *pink*. MTPs are labeled with system name, protein name, and Protein Data Bank ID, and, in the case of T6SS, the bacterial host organism. GTA, gene transfer agent; MTP, major tail protein; T6SS, type VI secretion system.

The tail tube of the bacteriophage-like GTA of *Rhodobacter capsulatus* belongs to the *Siphoviridae*-like tail morphotype. Compared with all other systems, it exhibits the shortest tail by only consisting of five rings with a thickness of 38.3 Å and a rotation toward each other by 24.4°. Each ring consists of six MTP Rcc01691 subunits. The cryo-EM map of the tail tube of GTA was helically reconstructed from images of a full GTA particle to a resolution of 3.6 Å. The MTP shows no structural changes upon DNA release, which highlights the role of the MTP merely being a scaffolding protein (76). The MTP of GTA has the most simple architecture of all MTPs described in this article—meaning the least amount of extra loop/linker regions in addition to the fold common to all MTPs. Hence, the basic building block of an MTP is demonstrated with this system (Fig. 3). One MTP subunit consists of a β-sandwich–type fold that is flanked by an α-helix. The β-strands are named and numbered in their sequential order β1, β2, β3, β4, β5.1, β5.2, β6.1, and β6.2. The inner β-sheet of the β-sandwich–type fold is formed by β2, β3, β6.1, and β5.2, and the outer β-sheet by β6.2, β5.1, β4, and β1 (going from left to right, with the α-helix oriented vertically on the right, outer side). β5.1 and β5.2, as well as β6.1 and β6.2, form semicontinuous and bent β-strands in some MTP systems (Fig. 3*A*). In addition, the MTP contains one long loop β2–β3. Six MTP monomers assemble to form a hexameric ring by creating an extensive β-barrel that lines the inner lumen of the tail tube, which has an approximate diameter of 40 Å. Herein, the β-barrel is formed by the inner β-sheet, and the α-helices are arranged parallel to the tail tube axis (Fig. 3*B*). These rings stack onto each other in a helical fashion creating a hollow tube. Interring contacts are herein mostly mediated by the loop β2–β3 and to a small extent by the N terminus (Fig. 3*C* and Table 2). A nomenclature of subunit naming within the tail tube complex can be appreciated in Figure 3*D*. Because of its simplicity, all other systems are compared with the MTP of GTA as visualized in Figures 4 and 5, where structural differences are highlighted in color.

### T5 phage tail tube—*Siphoviridae*-like

T5 phage belongs to the *Caudovirales* family of *Demerecviridae* and, hence, belongs to the *Siphoviridae*-like tail morphotype. However, compared with all other MTP systems, it has a unique tail tube organization: It uncommonly exhibits a trimeric ring—as opposed to the usual hexameric ring, which results from the fusion of every two subunits (77). Such an organization is further only known from the φCbK phage, which belongs to the *Siphoviridae* family (78). Hence, the trimeric nature of the tail tube does not seem to be confined to only one family. The tail tube consists of 40 trimeric rings with a thickness of 40.6 Å that stack onto each other rotated toward each other by 39.1°. It is assembled around a tape measure protein. The structure of pb6—the MTP of T5 phage—could be solved by X-ray crystallography at a resolution of 2.2 Å. In addition, a 6.2 Å cryo-EM map of the full tail tube—in the context of a full tail including the tail tip/tape measure protein—and 5.8 Å cryo-EM map of the empty tail tube (after receptor binding), and a 8.8 Å cryo-EM map of heterogeneously expressed pb6 tubes could be reconstructed. The heterogeneously expressed pb6 tubes only form spontaneously during cell lysis. Merely high concentrations of monomeric pb6 are not sufficient to induce self-polymerization as some trigger seems to be necessary for the induction of this polymerization event. However, general polymerization was noted to be enhanced at high salt and low pH conditions. At aforementioned resolutions, no structural differences of the tail tubes could be observed. The cryo-EM map of the full tail tube allowed for the generation of a pseudoatomic model of the T5 tail tube by modeling monomeric pb6 into the density. Based on this model and sequence alignments, pb6 can be divided into two domains. The N-terminal domain 1 to 374 contains the duplicated MTP domain, and the C-terminal domain contains an Ig-like fold of the Big_2 family, which protrudes into the exterior of the tail tube (Fig. 4). The tail tube itself is solely formed by the duplicated MTP domain (hereafter, MTP and MTP∗), which as usual consists of a β-sandwich–type fold that is flanked by an α-helix and the long loop β2–β3. Intraring contacts are mostly established by the formation of the 24-sheet β-barrel, which lines the inner lumen of the tube. Interring contacts are mostly established by the loop β2–β3 and loop β2–β3∗ and the N terminus. It must be noted here that the total per ring interaction surface of Table 2 only corresponds to three times this value—as opposed to six times for the usual hexameric-ringed systems. Mutation studies could show that the C-terminal Ig-like domain is indispensable for tube formation and acts as an independent unit from the tail, which could also be shown by solid-state NMR spectroscopy (79). Also, deletion of the N terminus, loop β2–β3, and loop β2–β3∗ completely abolish tube formation proving their importance in establishing intermolecular contacts. Further point mutations of conserved amino acids only decrease polymerization efficiency indicating that the general structure can cope with few local structural disruptions (80), which might promote rapid evolution.

### λ phage tail tube—*Siphoviridae*-like

λ phage belongs to the *Caudovirales* family of *Siphoviridae* and, hence, belongs to the *Siphoviridae*-like tail morphotype. The tail tube of λ phage consists of 32 stacked hexameric rings assembled around a tape measure protein (32). The rings have a thickness of 42.8 Å and are rotated toward each other by 17.5° (81). It was shown that the tail disassembles reversibly at pH < 2.8 or pH > 11.8 and reassembles upon return to neutral conditions. However, this disassembly at extreme pH values can be entirely suppressed by high salt conditions (>0.7 M NaCl) highlighting the importance of electrostatic interactions within the tail structure (82). The tail tube protein of this system is gpV, which consists of an N-terminal MTP domain (1–156) and a C-terminal domain (157–246) that is homologous to an Ig-like fold of the Big_2 family. Both monomeric substructures (hereafter, gpV_N_ and gpV_C_) were independently solved by solution NMR spectroscopy (83, 84). The solution NMR structure of gpV_N_ encompasses an α-helix and seven β-strands that fold into a bent β-sandwich fold—reminiscent of the typical MTP fold. In addition, the structure features three unfolded loop regions with dynamics on the ps–ns timescale (1–14, 50–78, and 149–153). Herein, the first region relates to the N-loop and the second region to the loop β2–β3. The structure also features a prolonged loop between β1 and β2 compared with other systems (hereafter, loop β1–β2; Fig. 4). The mutation of two conserved aspartate residues in loop β2–β3 to alanine (D61A/D62A) inhibits tail tube growth at the ring-to-ring interface highlighting the importance of electrostatic interactions within this interface (83). The solution NMR structure of the C-terminal domain gpV_C_ resembles a canonical Ig-like β-sandwich with two four-stranded β-sheets and shows that conserved residues are found either at the proximal (close to the MTP) or at the distal end of the fold. Also, it could be shown that the removal of the C-terminal domain decreases biological activity by 100-fold but maintains the ability to form tails (84). This is different to other MTP systems, where the removal of the Ig-like fold does not affect biological activity. Cryo-EM maps of native tails before and after receptor binding (“empty”) and a cryo-EM map of heterogeneously expressed free tail could be reconstructed to a resolution of 6.8, 6.4, and 5.4 Å focusing on the inner tube structure, respectively (the C-terminal protruding into the exterior of the tube was reconstructed separately). To model the structure of gpV within the context of the assembled tube, the solution NMR structures of gpV_N_ and gpV_C_ were fitted into the latter cryo-EM maps. Hereby, flexible fitting of gpV_N_ is necessary to accommodate the N-loop, loop β2–β3, and C terminus—the regions that showed elevated dynamics within the monomer. The Ig-like fold protrudes into the exterior of the tube oriented parallel to the tail tube axis. Herein, the Ig fold contributes slightly to the intraring contact I − (±1) by interacting with the loop β1–β2 of the next adjacent MTP. This is a difference to T5 phage tail tube where the Ig fold loosely protrudes into the exterior oriented orthogonal to the tail tube axis, which does not allow for any Ig-like fold–MTP contacts. This Ig-like fold arrangement is proposed to form a network on the surface of the tail tube that might be realized by the upper and lower ends of this domain having opposite polarity. However, this remains a suggestion as the domains do not bear any interfaces between each in the presented tail tube structure. Nevertheless, these regions are poorly resolved in the cryo-EM density resulting from flexibility, which might accommodate transient interactions of this type. Inner ring contacts in λ phage tail tube are mostly mediated by the formation of a 24-stranded β-barrel, whereas interring contacts are mostly mediated by the N-loop and loop β2–β3—both bearing a high local concentration of charged amino acids. This gives the tail tube a certain polarity by having the proximal ring-to-ring interface positively charged and the distal one negatively charged. The abundance of electrostatic interactions in the ring–ring interface in this system explains the ring dissociation upon extreme pH values. The inhibition of this effect at high salt concentrations possibly stems from charge masking. Unusually, the inner lumen of this tail tube bears both negatively and positively charged surfaces (81).

### YSD1 phage tail tube—*Siphoviridae*-like

YSD1 phage belongs to the *Caudovirales* family of *Siphoviridae* and, hence, belongs to the *Siphoviridae*-like tail morphotype. The tail of YSD1 phage was reconstructed helically from cryo-EM images of the full virion yielding a 3.5 Å cryo-EM map. The MTP YSD1_22 forms hexameric rings with a thickness of 42.7 Å that stack on each other rotated toward each other by 19.7°. The MTP is arranged in the canonical fashion. However, it features three additional domains in comparison to the GTA MTP: An N-loop (extension of the N terminus), an additional inserted domain, named domain2, and a C-terminal Ig-like domain of the Big_1 family. Domain2 is inserted between α1 and β4 and resembles a β-sandwich fold. This domain folds onto the outer β-sheet of the β-sandwich of the adjacent i + 1 MTP within the ring creating a huge additional interface for this type of contact stabilizing the hexameric unit. Similar to T5 phage, the C-terminal Ig-like domain merely loosely protrudes into the exterior oriented orthogonally to the tail—highlighted by its weak EM density signifying its flexibility and/or nonstrict helical symmetry. Also, the YSD1 tail tube lumen has an unusual hexagonal profile because of the residues 240 to 242 that form a stagger that protrudes into the interior of the tail tube. The inner lumen of the tube exhibits a negative electrostatic potential and negatively charged Asp and Glu residues oriented in a fashion that might promote the conduit of the DNA through the tail passage. Recombinantly expressed YSD1_22 remains monomeric in solution. Analysis by small-angle X-ray scattering revealed a low-resolution model that accounts for most of the structure of polymerized YSD1_22. However, the N-loop and loop β2–β3 are not accounted for, and are, therefore, probably highly dynamic in the context of monomeric MTP species. Recombinantly expressed MTP without the N-loop remains monomeric, whereas removal of the loop β2–β3 leads to the formation of oligomers—mostly hexameric rings. This contributes two possible functions to the loop β2–β3 in this system: inhibition of monomeric MTPs to form hexameric rings in the absence of the initiation complex and mediation of inter-ring contacts in the polymerized tail tube (85).

### 80α phage tail tube—*Siphoviridae*-like

80α phage belongs to the *Caudovirales* family of *Siphoviridae* and, hence, belongs to the *Siphoviridae*-like tail morphotype. A cryo-EM map of the baseplate of 80α phage was reconstructed to a resolution of 3.7 Å, which includes two rings of the tail tube of that system. Here, these two last rings of the tail tube are anchored within the baseplate complex—and are bundled similarly to the tail tube by the sheath protein in the case for the *Myoviridae* morphotype, and are, hence, not part of the flexible region of the tail tube. Nevertheless, the first 3 and 24 last residues are not part of the final structure—probably because of insufficient resolution in the periphery of the tail tube. About 39 rings of hexameric rings, which consist of six gp53 subunits, stack onto each other creating a hollow tube. gp53 features the usual MTP fold but has two additional linker regions: the loop β4–β5.1 and C-arm. The C-arm is a C-terminal extension that folds onto the outer β-sheet of the superjacent subunit expanding the inter-ring contacts in this system. gp53 has no C-terminally attached Ig-like fold. The inner lumen of the 80α phage tail tube is electrostatically negatively charged (20).

### SPP1 phage tail tube—*Siphoviridae*-like

SPP1 phage belongs to the *Caudovirales* family of *Siphoviridae* and, hence, belongs to the *Siphoviridae*-like tail morphotype. The tail tube of SPP1 phage consists of approximately 40 stacked hexameric rings assembled around a tape measure protein (26). The rings have a thickness of 38.5 Å and are rotated toward each other by 21.9° (86). The MTPs of this system are gp17.1 and gp17.1∗—the latter being a C-terminally extended variant because of a translational frameshift—and occur in a ratio of 3:1. This C-terminal extension is an FN3 domain, which resembles an Ig-like fold. However, it was shown that this domain is not necessary for phage infection and viability (22). At concentrations of 100 μM, gp17.1 remains monomeric and can be studied by liquid-state NMR. The monomeric structure in solution features as the lone observable secondary structure part of the β-sheet formed by β3, β6.1, and β5.2, the rest of the protein is in conformational exchange—with chemical shifts indicating random coil structure. Upon concentration to 400 μM, gp17.1 self-polymerizes into infinitely long tubes that are indistinguishable from native tail tubes as judged by negative-stain EM. This polymerization goes in hand with an increase of β-strand secondary structure—most likely because of the formation of the rest of the β-sandwich and β-barrel (33). The structure of the heterogeneously expressed tail tube of SPP1 phage was solved in a hybrid approach integrating a 4.3 Å resolved cryo-EM map with distance restraints from solid-state NMR simultaneously. This allowed for the determination of the structure of two rings of the tail tube at a resolution of 1.8 Å. The MTP gp17.1 features a structure very similar to gp53: The standard MTP fold, and in addition, a prolonged C-arm and a loop β4–β5.1. However, the C-arm is entirely resolved in this structure since the lack of resolution in the cryo-EM map in the periphery of the tail tube can be compensated for by NMR restraints. The C-arm folds onto the superjacent subunit by anchoring a Gln residue in a pocket and interacting with a hydrophobic patch on the outer β-sheet of monomeric gp17.1. The inner lumen of the tail tube carries a negative electrostatic potential suitable for DNA conduit. In addition, elevated dynamics on the nanosecond–microsecond timescale, as judged by relaxation measurements, could be associated with variances in the cryo-EM map. These regions were also identified as hinge regions in a model of the bending of the tail tube—highlighting their role as driving forces for the flexibility of this system (86). Removal of the loop β2–β3 or the C-arm inhibits self-polymerization of monomeric MTP but keeps the monomeric fold similar to wt monomeric gp17.1 (33, 87).

### T6SS tail tube—*Myoviridae*-like

The Hcp1 protein of the T6SS of *Vibrio cholera* belongs to the *Myoviridae*-like tail morphotype. The cryo-EM structure of the T6SS tail tube was solved as part of the extended type VI secretion system sheath–tube complex at a resolution of 3.7 Å. The tail tube rings have a thickness of 37.8 Å and are rotated toward each other by 23.5°. The MTP Hcp1 features four differences compared with the basic MTP of GTA: the loop β5.2–β6.1; the loop β1–β2; the loop β4–β5.1; and a C-terminal extension of the outer β-sheet, namely β-sheet ext. The latter three additional features can also be appreciated in crystal structures of T6SS Hcp1 and crystal structures of further systems (Fig. 5). Unusually, the inner diameter of the tail tube fluctuates between 20 and 35 Å because of the orientation of the loop β5.2–β6.1 that protrudes into the interior of the tail tube lumen (88).

### T4 phage tail tube—*Myoviridae*-like

T4 phage belongs to the *Caudovirales* family of *Myoviridae* and, hence, belongs to the *Myoviridae*-like tail morphotype. The cryo-EM structure of two rings of the T4 phage tail tube were solved as part of the prehost-attachment baseplate of T4 phage at a resolution of 3.8 to 4.1 Å (47), and, in addition, a reconstruction exclusively focused on the tail tube based on the same micrographs yielded a resolution of 3.4 Å (50). The hexameric rings have a thickness of 40.2 Å and are rotated toward each other by 17.9° (47). The MTP gp19 features three differences compared with the basic MTP of GTA: the N-loop and β4–β5.1, which are also present in some *Siphoviridae*-like systems, and the α-loop. These additional loops allow for extensive protein–protein contacts. The inner lumen of the tail tube carries a negative electrostatic potential suitable for DNA conduit (50).

### PVC—*Myoviridae*-like

The protein Pvc1 of the PVC belongs to the *Myoviridae*-like tail morphotype. The structure of this phage-like protein-translocation structure was solved by cryo-EM and contains a phage-like tail tube at a resolution of 2.9 Å. The tail tube of the PVC particle consists of 22 stacked hexameric rings of Pvc1. The hexameric rings have a thickness of 39.3 Å and are rotated toward each other by 19.9°. The tail tube bears a certain polarity by having the proximal ring-to-ring interface positively charged and the distal one negatively charged. Pvc1 has three additional features compared with the MTP of GTA: the N-loop; β4–β5.1 loop; and α-loop. The weak tube–sheath interaction (Table 2) compared with the tube–tube interaction allows for sliding of the sheath along the tube upon contraction. The inner lumen of the tail tube of PVC carries a negative electrostatic potential (89).

### AFP—*Myoviridae*-like

The protein Afp1 of AFP of *Serratia entomophila* belongs to the *Myoviridae*-like tail morphotype. The structure of the MTP Afp1 of this phage-like protein-translocation structure was solved by cryo-EM as part of the trunk—containing tail tube and sheath—in the extended state at a resolution of 2.8 Å. The tail tube of the AFP particle consists of 21 stacked hexameric rings of Afp1. The hexameric rings have a thickness of 39.3 Å and are rotated toward each other by 20.1°. Afp1 features the same additional domains as gp19 and Pvc1: the N-loop; β4–β5.1 loop; and α-loop. Similar to the previous case, the tube–sheath interaction is weak compared with the tube–tube interaction (Table 2). The inner lumen of the tail tube of AFP carries a negative electrostatic potential (75). This is suitable to conduct the toxin Afp18, which is negatively charged in the gut of *Costelytra giveni* larvae (90). The most proximal hexameric ring of Afp1 is structurally slightly different compared with the other rings. Its α-helix in the N-loop unwraps to interact with the cap forming protein Afp16.

### Pyocin R2—*Myoviridae*-like

The protein Pa0623 of the phage tail–like bacteriocin pyocin R2 of *Pseudomonas aeruginosa* belongs to the *Myoviridae*-like tail morphotype. The structure of the MTP Pa0623 of this phage-like protein-translocation structure was solved by cryo-EM as part of the trunk—containing tail tube and sheath—in the extended state at a resolution of 2.9 Å (70). The tail tube of the pyocin R2 particle consists of 28 stacked hexameric rings of Pa0623. The hexameric rings have a thickness of 38.4 Å and are rotated toward each other by 18.3°. The tail tube bears a certain polarity by having the proximal ring-to-ring interface negatively charged and the distal one positively charged (69). Pa0623 carries as additional structural features the N-loop, β4–β5.1, and β-sheet ext. Compared with the T6SS systems, the β-sheet extension of Pa0623 is the most extended, even carrying an additional α-helix at the very C terminus (70). The structure of the tail tube after sheath contraction is unknown, since the helical symmetry of the sheath does not correspond to that of the tube afterward. This is possible because of the tail tube being able to move more freely after sheath contraction and expansion, that is, it is detached from the sheath. No reconstruction of this state solely focused on the tail tube was conducted (69). The inner lumen of the tail tube carries nearly equally negative, positive, and neutral electrostatic potentials, which highlights the role of the phage tail–like bacteriocin being merely active by puncturing the host's membrane, that is, the tail tube does not conduct any cargo (50).

## Comparison of structural features

All described MTPs share a β-sandwich-like fold, which is flanked by an α-helix, and the loop β2–β3. The inner β-sheet of the β-sandwich forms a 24-stranded β-barrel that lines the inner lumen of the tail tube. This represents the largest intraring contact for all systems—except for YSD1 phage that features the additional domain2 embracing the neighboring i + 1 subunit (85). For bacteriophages, the electrostatic potential of the inner lumen of the tail tube carries a negative charge suitable for the transport of DNA—with the exception of λ phage, which displays positive and negative charges (81). For PVC, the negative electrostatic potential of the lumen also matches the charge of its cargo at physiological conditions (89).

The loop β2–β3 conveys the largest inter-ring contact for the described systems—except for SPP1 phage (86) and 80α phage (20), which feature the C-arm that folds onto a subjacent subunit. Deletion of the loop β2–β3 abolishes tail tube formation in T5 phage (80), and more precisely of tail tube growth at the ring-to-ring interface in SPP1 phage (33), which can also be obtained in λ phage by mutation of two electronegative residues to alanine within this loop (83). In YSD1 phage, deletion of this loop leads to the formation of hexameric rings (85). In monomeric MTP, the loop β2–β3 is highly dynamic as shown for λ, SPP1, and YSD1 phages (33, 83, 85). Hence, loop β2–β3 could encompass a bimodal function: inhibition of monomeric MTP in solution to form hexameric rings and mediation of inter-ring contacts by electrostatic interactions (81). However, the polarity of this electrostatic interaction is not conserved as it appears to be mostly negative for λ phage (81) and PVC (89) and positive for pyocin R2 (69).

The C-terminal Ig fold (FN3 for SPP1 phage (22), Big_1 for YSD1 phage (85), Big_2 for λ (84), and T5 phage (80)) is only present in some *Siphoviridae*-like systems since they would clash with the sheath protein in *Myoviridae*-like systems. For YSD1 (85) and T5 phages (80), this domain merely flexibly protrudes from the outer surface of the tail tube oriented orthogonally to the tail tube axis. Here, the Ig-like domain is proposed to aid the infection process by weakly interacting with carbohydrates on the surface of bacteria (23), which remains to be experimentally verified. In contrast for λ phage, this fold seems to play a more intricate role: The domain protrudes from the outer surface of the tube oriented parallel to the tail tube axis—forming a network on the surface of the tail tube (81). Herein, it contributes an additional intraring contact, and removal of it leads to a decrease in phage activity (84). This is different to SPP1 phage, where this removal is irrelevant for infection and phage viability (22)—underlining the contrasting role of the domain.

The N-loop exists for the *Siphoviridae*-like λ phage and YSD1 phage and for all *Myoviridae*-like systems except for T6SS systems. In monomeric λ (83) and YSD1 phage, this loop is highly dynamic, and removal of it in YSD1 phage inhibits tail tube association (85). This highlights its importance as a protein–protein interface—mostly interacting with the loop β2–β3. In all described *Myoviridae*-like systems with the exception of AFP, the N-loop also contacts the outer sheath proteins.

The C-arm has so far only been found in 80α (20) and SPP1 phage (86) and, thus, might be a structural feature of a subgroup of the *Siphoviridae* family. It is found in the C terminus and folds onto the superjacent subunit promoting the largest inter-ring contact in these systems. In both systems, it folds onto the outer β-sheet of the β-sandwich fold. Removal of the C-arm in SPP1 phage inhibits phage tail polymerization (87). This is because of a hydrophobic patch on the outer β-sheet of one gp17.1 subunit that is obscured by the C-arm (86).

Loop β4–β5.1 is found in the *Siphoviridae* 80α (20) and SPP1 phage (86) where it does not contribute to any intermolecular contacts but loosely protrudes into the exterior of the tail tube. In SPP1, this structural feature displays dynamics on the nanosecond timescale as judged by NMR relaxation analysis (86). Note, that the other described systems have so far not been investigated in terms of dynamics by NMR spectroscopy. In *Myoviridae*-like systems, all but one MTP features this domain. Here, it is a main contributor to the tail tube–sheath interface.

In λ phage, loop β1–β2 contributes slightly to an intraring contact by interacting with the C-terminal Big_2 domain (81). In the T6SS of *Vibrio cholera*, this loop interacts with the outer sheath (88).

The α-loop occurs in all non-T6SS *Myoviridae*-like systems and forms an intermolecular bridge between the sheath, N-loop, and loop β2–β3 for AFP (75) and PVC (89). However, this cannot be fully appreciated in T4 phage because of the missing of the sheath in the structure.

β-sheet extension is present in all T6SS systems and pyocin R2 (70). The outer β-sheet of the β-sandwich fold of *Myoviridae*-like MTP is a major contributor to the tail tube–sheath interaction. Hence, the β-sheet extension expands this outer β-sheet allowing for a more intricate tail tube–sheath interface.

## Tail tube flexibility

Protein flexibility describes the possibility for a biological system to dynamically sample a continuous set of structures within a certain range of structural extremes and timescales. The flexibility of bacteriophage tail tubes more generally refers to the bending ability of these systems. On a local level, this bending requires the compression or stretching of certain subunits within the tail, which in turn requires disorder or local structure disruptions by protein dynamics. It is expected that a reduced interaction interface between MTP subunits facilitates this process as it allows for more local degrees of freedom. Table 2 shows that *Siphoviridae*-like tail tubes trend toward smaller per ring protein–protein interfaces than *Myoviridae*-like tail tubes—which also bear the tube–sheath interfaces that in addition bundle the tail tube. Hereby, YSD1 phage marks an exception, whose additional domain2 encodes a huge extra inner-ring interface. However, tails of YSD1 phage and χ phage—a closely related phage carrying the same additional domain2—appear rather straight in negatively stained electron micrographs, and might mark an exception for this phage family (85, 91).

The resolutions in cryo-EM maps appear to follow a similar trend as shown in Table 3. More flexible *Siphoviridae*-like tail tubes trend toward lower resolved cryo-EM maps.

**Table 3:** Cryo-EM resolutions of bacteriophage tail and bacteriophage tail–like MTPs

*Siphoviridae*-like			*Myoviridae*-like		
System	Year	Resolution in Å	System	Year	Resolution in Å
T5 phage	2017	6.2	T6SS	2017	3.7
λ phage	2019	5.4	T4 phage	2017	3.4
GTA	2020	3.8	PVC	2019	2.9
YSD1 phage	2020	3.5	AFP	2019	2.8
80α phage	2020	3.7	Pyocin R2	2020	2.9
SPP1	2020	4.3			
		⌀ 4.5 ± 0.8			⌀ 3.1 ± 0.3

The arithmetic mean and mean deviation of the cryo-EM resolutions of *Siphoviridae*-like and *Myoviridae*-like systems are compared.

**Figure 6:**
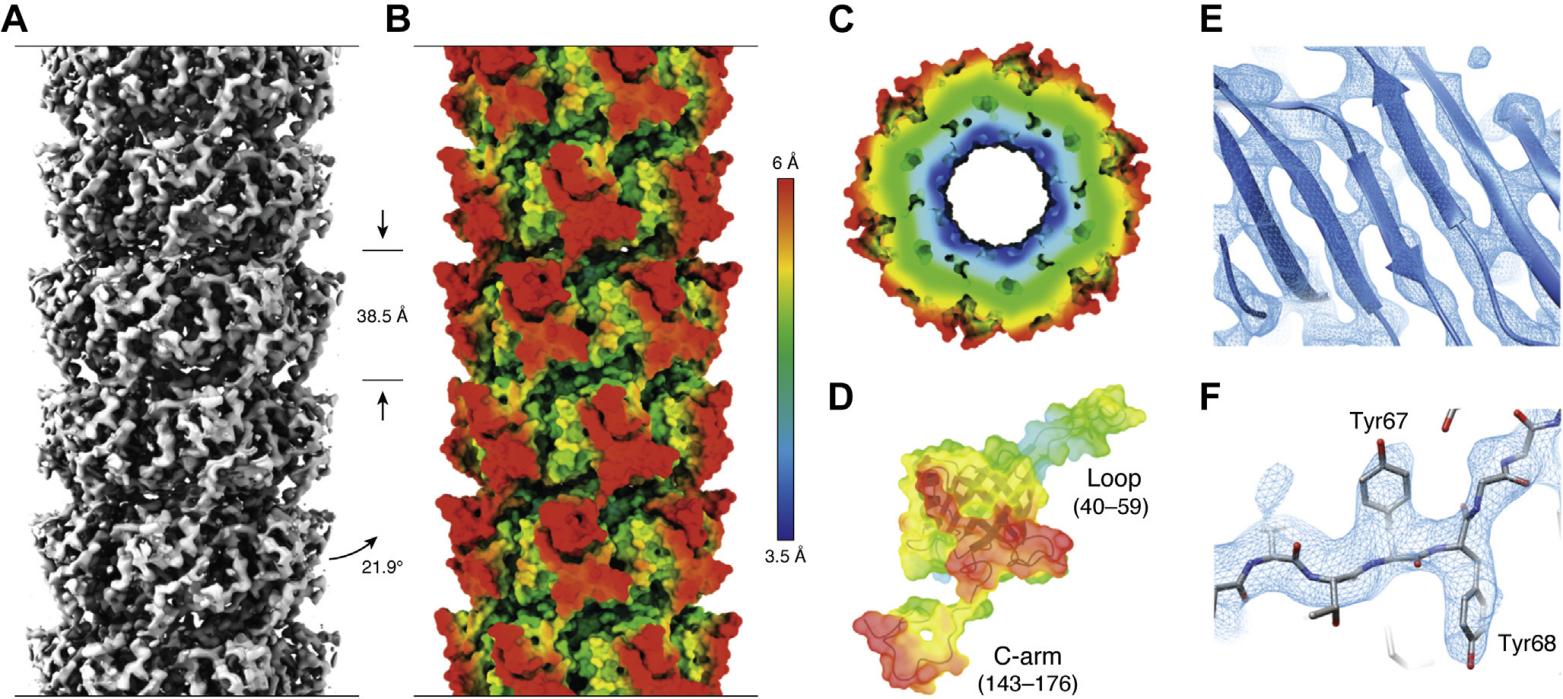
**The cryo-EM density of the tail tube of SPP1 phage.***A*, displays a local resolution distribution from 3.5 to 6 Å. High resolution at the inner surface of the tail tube allows for a straightforward positioning of the bulky amino acid side chains. However, the lack of resolution at the periphery required additional structural restraints from, in this case, solid-state NMR for a structure calculation following an integrative approach (*B*–*F*). Reproduced with minor changes under a Creative Commons Attribution 4.0 International License (http://creativecommons.org/licenses/by/4.0/) from the study by Zinke *et al.* (86).

For SPP1 phage, this heterogeneity manifests as wide distribution of local resolutions and variances in the cryo-EM density. As shown in Figure 6, the local resolution of the cryo-EM density map decreases going from inner to the outer surface of the tail tube—making a confident structure calculation only possible in an integrative approach by combining these cryo-EM data with restraints from solid-state NMR. Regions with pronounced variances show stronger dynamics on the nanosecond–microsecond timescale as probed by NMR and could be identified as hinge regions during the bending process of the tail tube—which itself happens on the microsecond timescale (86). Hence, local dynamics on the nanosecond–microsecond timescale might prompt local structure disruptions, which manifest themselves as continuous heterogeneity in cryo-EM maps and, thus, a lack of resolution. In addition to decreased subunit–subunit interface area, these dynamics might give the system degrees of freedom to allow for tail tube bending. A similar limitation of the cryo-EM resolution can be appreciated by the structural biology efforts on the type 2 secretion system and the type 4 pilus, where structural heterogeneity prevented high-resolution reconstructions (92, 93, 94, 95, 96). In the type 2 secretion system, dynamics on the ps–ns timescale were proposed to be responsible for this structural heterogeneity. Nevertheless, there are three *Siphoviridae*-like systems that do not follow this trend and show cryo-EM resolutions sub 4 Å: GTA, YSD1 phage, and 80α phage. For GTA, the tail tube only consists of five hexameric rings of MTP (76). This makes the entire tail very compact and might allow for less flexibility. YSD1 phage on the other side bears the additional domain2, which allows for the second largest subunit–subunit interface, and bears seemingly rather straight tails (85, 91). For 80α phage, the tail tube structure of two rings embedded in the baseplate of these systems was analyzed, which corresponds to an additional bundling—similar to the sheath of *Myoviridae*-like tail morphotypes, and does hence not reflect the flexible part of this tail tube (20).

## Outlook

The structural biology effort on MTPs of bacteriophages and similar systems in the last years has provided a deep insight into the tertiary and quaternary structures of these systems. In this review, we have summarized their structural elements showing that some are constant and others are variable. The ongoing methodological progress in cryo-EM and NMR spectroscopy—like the recently commercially available 1.2 GHz NMR spectrometer—will continue to make structures of such type more accessible. This could reveal more variable elements within MTPs and might allow for family-specific classification of these—especially since MTP structures of some families are not available yet (*e.g.*, for *Drexlerviridae* of the *Siphoviridae*-like phages and for *Ackermannviridae*, *Chaseviridae*, and *Herelleviridae* of the *Myoviridae*-like phages).

Hardy *et al.* (85) have assigned negatively charged residues oriented in a certain fashion within the inner lumen for the promotion of DNA conduit through the tail tube. Such DNA/cargo–MTP interaction studies could be greatly expanded to further systems—even to tape measure protein–MTP interactions—and might allow for the understanding of the molecular processes of the infection process of phages. Also, the biological role of the Ig folds that are found on the outside of some of the described *Siphoviridae*-like tail tube systems remains to be determined. So far, it is only speculated that they might facilitate the infection process by interacting with carbohydrates on the bacterial surface (23). It is also unclear if they are just positioned loosely into the environment or if they bear a higher-ordered structure.

The nonflexible *Myoviridae*-like phages use a syringe-like mechanism to drive their tail tube through the outer membrane of host bacteria. This physical piercing process is expected to require a certain nonflexibility of the tail. On the contrary, the flexible *Siphoviridae*-like phages do not utilize such a mechanical piercing mechanism for infection—at least to our current knowledge. It is proposed that their ejected tape measure protein merely creates a pore in the host membrane for DNA translocation (35), and, hence, these phages might not bear the necessity of a rigid tail. Maybe, flexibility of the phage tail allows for a more efficient screening of host receptors.

Further assessments of the flexibility and dynamics of these systems—ideally in a combined approach of cryo-EM, NMR, and molecular dynamics simulations—will have to give a more detailed insight into these effects and might explain what biological processes are influenced by increased flexibility. Also, electron cryotomography of phages infecting bacteria will prove crucial to describe the entire infection process on a molecular level.

## Conflict of interest

The authors declare that they have no conflicts of interest with the contents of this article.
